# Reactive oxygen species mediate ovarian cancer development, platinum resistance, and angiogenesis via CXCL8 and GSK-3β/p70S6K1 axis

**DOI:** 10.1016/j.gendis.2024.101378

**Published:** 2024-07-17

**Authors:** Jiange Qiu, Qing Xu, Tahira Panah, A K M Helal Morshed, Xue Wang, Fengmei Zhou, Wenjing Liu, Jufeng Wang, Ye Zhang, Bingjie Liu, Bing-Hua Jiang

**Affiliations:** a The First Affiliated Hospital of Zhengzhou University, The Academy of Medical Science, Zhengzhou University, Zhengzhou, Henan 450000, China; b Department of Pathology, Nanjing Medical University, Nanjing, Jiangsu 210029, China; c The Affiliated Cancer Hospital of Zhengzhou University, Academy of Medical Science, Zhengzhou University, Zhengzhou, Henan 450000, China

Ovarian cancer is one of the leading causes of cancer-related deaths in women worldwide, with a five-year overall survival rate of less than 50% or 30% if it is in the advanced stages.^1^ Reactive oxygen species (ROS) are typically involved in signal transduction and gene activation.^2^ Recent studies suggest increased levels of ROS in cancer development,^3^^,^^4^ but the role and mechanism of ROS in ovarian cancer angiogenesis and development remain to be elucidated. Our study found much higher levels of ROS and C-X-C motif chemokine ligand 8 (CXCL8) in ovarian cancer cells and tumor tissues. ROS-induced CXCL8 up-regulation through glycogen synthase kinase-3 beta (GSK-3β) inactivation and p70S6 kinase 1 (p70S6K1) activation. There were strong positive correlations between expression levels of CXCL8 and p-GSK-3β/p-p70S6K1, showing that p-GSK-3β and p-p70S6K1 levels were important in CXCL8 expression in ovarian cancer. Furthermore, inhibition of p70S6K1 using its inhibitor PF-4708671 greatly increased the cytotoxic effect of platinum. ROS and CXCL8 are potential new therapeutic target(s) and biomarker(s) for ovarian cancer development in the future.

We measured ROS and CXCL8 levels in serum and tissue samples from ovarian cancer patients, benign tumors, and healthy subjects. Our results showed that ROS levels in malignant ovarian tumors were significantly higher than in the other two groups (Fig. 1A; Fig. S1A). CXCL8 levels were significantly higher in serious cancer patients (Fig. S1B, C). Next, we analyzed the correlation between serum CXCL8 and ROS levels of cancer tissues, and found a significantly positive correlation between their levels (Fig. 1B). For the survival analysis, higher ROS levels showed lower survival rates in ovarian cancer patients (Fig. 1C). Similarly, higher CXCL8 levels were correlated with poor outcome (Fig. S1D, 2). These results demonstrate that high levels of serum CXCL8 and ROS are significantly associated with a poor prognosis in ovarian cancer patients.

**Figure 1:**
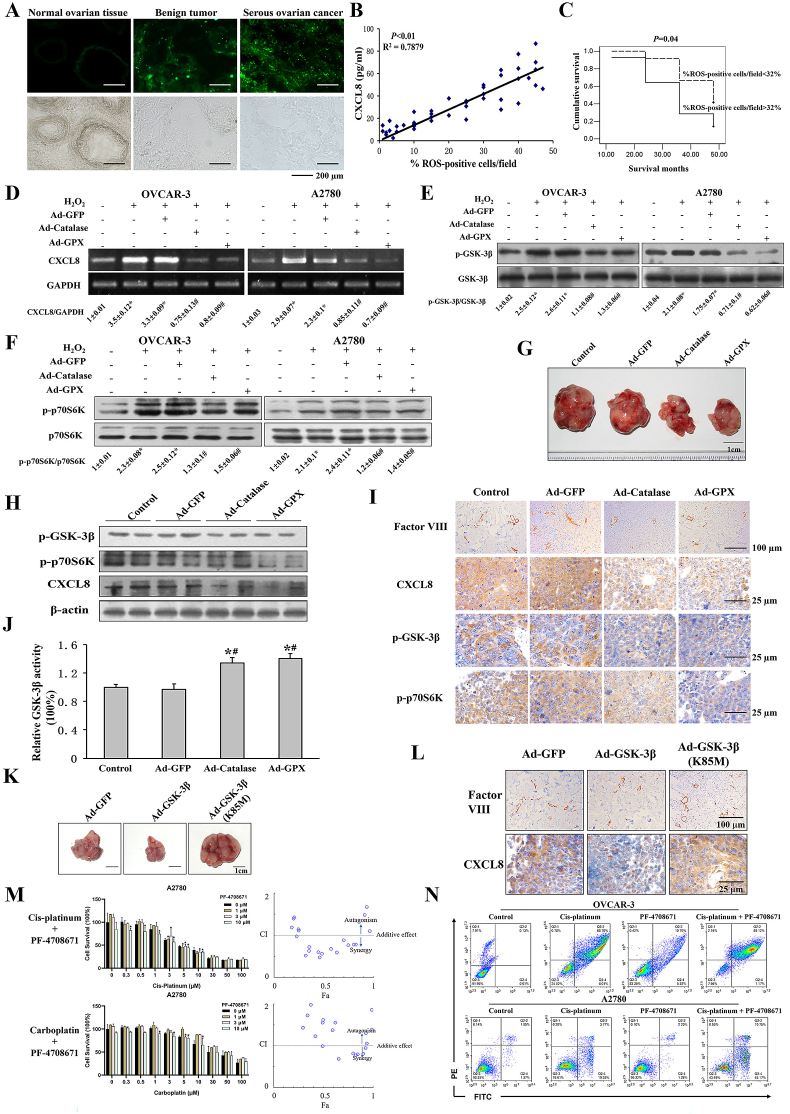
ROS mediate ovarian cancer development, platinum resistance, and angiogenesis via CXCL8 and GSK-3β/p70S6K1 axis. **(A)** Human ovarian cancer tumor samples and normal ovarian tissues were stained with CM-H2DCFDA. The levels of ROS in the cells of the normal ovary and serous ovarian carcinoma were monitored by microscopic analysis. Scale bar, 200 μm. **(B)** The correlations between serum CXCL8 and ROS levels in ovarian cancer tissue and normal ovarian tissues were analyzed. **(C)** Kaplan–Meier survival analysis for %ROS-positive cells/field with cutoff 32% of ovarian cancer patients. **(D)** Cells were infected by adenovirus carrying GFP, catalase, and GPX, starved, and treated with H_2_O_2_ (100 μM). Levels of CXCL8 and GAPDH were measured using reverse transcription-PCR. The densitometry data represents the CXCL8/GAPDH signal ratios normalized to the control. **(E, F)** A2780 and OVCAR-3 cells were infected with or without adenovirus-carrying catalase, GPX, or GFP control. Serum-starved cells were treated with or without H_2_O_2_ (100 μM). The expression levels of (E) GSK-3β, and p-GSK-3β (Ser9) and (F) p70S6K and p-p70S6K (Thr389) were analyzed. **(G)** Representative tumors from each group. Scale bar, 1 cm. **(H)** Analysis of p-GSK-3β (Ser9), p-p70S6K (Thr389), and CXCL8 protein levels in each tumor by western blotting. **(I)** The expression levels of factor VIII, CXCL8, p-GSK-3β (Ser9), and p-p70S6K (Thr389) were detected using immunohistochemistry. **(J)** GSK-3β activities were determined using the GSK-3β activity assay kit. **(K)** Representative pictures of GFP, GSK-3β, and GSK-3β K85M groups. Scale bar, 1 cm. **(L)** Tumor sections from control and adenovirus-infected mice stained with factor VIII and CXCL8 antibodies. **(M)** A2780 cells were treated with indicated concentrations of PF-4708671 and cis-platinum/carboplatin, and cell survival rates were measured by CCK-8 assay after 72 h. Data were analyzed with CompuSyn software and the scattergram of combined effects was shown. **(N)** OVCAR3 and A2780 cells were seeded into 6-well plates and treated with indicated concentrations of PF-4708671 and cis-platinum, and the cell apoptosis levels were detected by Flow cytometer. ∗*P* < 0.05 compared with the solvent control; ^#^*P* < 0.05 compared with the group infected by GFP adenovirus. ROS, reactive oxygen species; CXCL8, C-X-C motif chemokine ligand 8; GSK-3β, glycogen synthase kinase-3 beta; p70S6K1, p70S6 kinase 1.

H_2_O_2_, a kind of ROS and well-characterized ROS inducer, significantly increased CXCL8 expression (Fig. S3A). Conversely, overexpression of catalase and glutathione peroxidase (GPX) abolished H_2_O_2_-induced CXCL8 mRNA expression (Fig. 1D). To further study the mechanism of ROS in inducing CXCL8 expression, we found that H_2_O_2_ treatment alone resulted in a significant increase in p-GSK-3β (Ser9) and p-p70S6K (Thr389) expression, whereas overexpression of catalase and GPX attenuated H_2_O_2_-induced p-GSK-3β (Ser9) and p-p70S6K (Thr389) expression (Fig. 1E, F). Furthermore, overexpression of GSK-3β inhibited ROS-induced CXCL8 expression (Fig. S3B). We also created a "kinase-dead" mutant form of GSK-3β (GSK-3β-KM (K85M)), and overexpression of GSK-3β-KM (K85M) increased CXCL8 levels (Fig. S3C). Exposing cells to LiCl, a GSK-3β inhibitor, yielded similar results to those for GSK-3β-KM (K85M) (Fig. S3D), indicating that GSK-3β inhibition induces CXCL8 expression. Furthermore, we found that overexpression of p70S6K increased CXCL8 levels (Fig. S3E). These results suggest that ROS regulates CXCL8 levels via GSK-3β and p70S6K in ovarian cancer cells.

We performed a tumor growth assay *in vivo* by comparing with the Ad-GFP group mice and showed that the tumor weights of the Ad-Catalase and Ad-GPX groups were much smaller (Fig. 1G; Fig. S4A). Overexpression of catalase or GPX inhibited angiogenesis with a significant reduction of hemoglobin levels (Fig. S4B). The expression levels of p-GSK-3β and p-p70S6K in tumor tissues with overexpression of catalase or GPX were greatly decreased (Fig. 1H, I; Fig. S4D–F). We also showed that decreased ROS levels caused by catalase or GPX overexpression resulted in much higher GSK-3β activities (Fig. 1J). Tumors generated from OVCAR-3 cells overexpressing GSK-3β were significantly smaller than the Ad-GFP control group. In contrast, tumor growth was dramatically increased in the GSK-3β-KM (K85M) group (Fig. 1K; Fig. S5A). These results suggest that down-regulation of ROS levels significantly inhibited tumor growth via GSK-3β and p70S6K.

To address whether GSK-3β is involved in tumor angiogenesis, tumor vascularization was quantified by Factor VIII and the hemoglobin content levels. Compared with the Ad-GFP control group, tumors overexpressing GSK-3β had less vascularization (12 versus 18 MVD/field) and lower hemoglobin content levels (1.92 versus 3 mg/g). In comparison, more vascularization signals (23 versus 18 MVD/field) and higher hemoglobin content levels (4.33 versus 3 mg/g) were detected in tumors overexpressing GSK-3β (K85M) (Fig. 1L; Fig. S5B, C). We found lower CXCL8 levels in tumors overexpressing GSK-3β, but higher CXCL8 levels in tumors overexpressing GSK-3β (K85M) compared with the control (Fig. 1L; Fig. S5D). These results show that GSK-3β inactivation promotes tumor angiogenesis. At the same time, higher protein levels of CXCL8, p-GSK-3β (Ser9), and p-p70S6K (Thr389) were found in ovarian cancer tissues (Fig. S6A, B). CXCL8 levels were positively correlated with levels of secretion CXCL8, p-GSK-3β (Ser9), and p-p70S6K (Thr389) (Fig. S6C–E). We showed that platinum combined with PF-4708671, a specific p70S6K1 inhibitor, had high drug synergy (Fig. 1M; Fig. S7A). Furthermore, the cell apoptosis results showed that the combination group showed higher levels of cell apoptosis levels (Fig. 1N; Fig. S7B). These data suggest that certain concentrations of PF-4708671 and cis-platinum/carboplatin had a drug synergy effect and enhanced cellular apoptosis.

In conclusion, our results demonstrate that high levels of ROS in ovarian cancer tissues lead to CXCL8 induction via activation of p70S6K1 and inhibition of GSK-3β. Levels of ROS, CXCL8, p70S6K1 activation, and GSK-3β inhibition are highly correlated with poor survival. The coordinated levels of ROS, p-GSK-3β (Ser9), p-p70S6K (Thr389), and CXCL8 from ovarian cancer may be used as new biomarkers for ovarian cancer development and prognosis. PF-4708671 combined with platinum may be used as a novel strategy to overcome ovarian cancer drug resistance in the future.

## Ethics declaration

All human tumor samples used in the study were from the tissue bank of Zhengzhou University without the patients' personal information. The Institutional Committee on Animal Care of Zhengzhou University approved the procedure of animal use (No. 2021-KY-0178).

## Author contributions

Conceptualization, B.H.J. and B.J.L.; methodology and investigation, Q.X., J.G.Q., M.A.H., P.T., X.W., F.M.Z., W.J.L., J.F.W., and Y.Z.; writing—original draft preparation, Q.X. and J.G.Q.; review and editing, B.H.J. and B.J.L.; project administration, B.J.L., Q.X., J.G.Q., and B.H.J.; bioinformatic analyses and data analyses, X.W. All authors read and agreed to the published version of the manuscript.

## Conflict of interests

The authors declared no competing interests.

## Funding

This work was supported by the National Natural Science Foundation of China (No. 81903174, 82073393), the Provincial Health Commission of Henan, China (No. SBGJ202003013, LHGJ20190656), the Medical Science and Technology Research Plan Provincial Co-construction Project of Henan Province, China (No. SBGJ202302023), and the Medical Science and Technology Project of Henan Province, China (No. SB201901110).
