# Supplementary material for: Reactive oxygen species mediate ovarian cancer development, platinum resistance, and angiogenesis via CXCL8 and GSK-3β/p70S6K1 axis
Source: Genes Dis. 2024 Jul 17;12(2):101378. doi: 10.1016/j.gendis.2024.101378 (PMC11629555; doi:10.1016/j.gendis.2024.101378)
Supplement: Multimedia component 1 [file mmc1.docx]

**Material and Methods**

**Clinical Patients and Tumor Tissues**

Ovarian tumor tissues, normal ovarian tissues, and blood samples of forty-four patients were obtained from Tissue Bank at the first Affiliated Hospital of Zhengzhou University. Histologically, 26 samples were diagnosed as ovarian cancer, 13 samples were benign tumors, and five were healthy ovarian tissues. The average age of patients with ovarian cancer and the control group was 55 years (39-82) and 49 years (36-64), respectively. The diagnoses and grading of all cases were peer-reviewed by two experienced pathologists according to the principles of the latest World Health Organization Classification. These samples were stored in the tissue bank with all cases classified and selected based on diagnosis using the CoPath Anatomic Pathology system and caBIG (The cancer Biomedical Informatics Grid). No HIPAA regulated information was included in the study.

**Antibodies and Reagents**

GSK-3β, p-GSK 3β (Ser9), p70S6K, p-p70S6K (Thr389), and mouse CD31 antibodies were purchased from Cell Signaling Technology (Danvers, MA). The CXCL8 antibody was purchased from Bioworld Technology Inc (Nanjing, China). Drabkin’s reagent kit and GSK-3β activity assay kit were purchased from MilliporeSigma (St. Louis, MO). CM-H2DCFDA was obtained from Thermo Fisher Sicientific (Waltham, MA). Taq polymerase, oligo(dT)18 primers, and Moloney murine leukemia virus (M-MLV) reverse transcriptase were from Takara (Beijing, China).

**Cell Culture**

Human ovarian cancer cell lines A2780 and OVCAR-3 were cultured in Dulbecco’s Modified Eagle Media (DMEM), supplemented with 10% fetal bovine serum (FBS), 100 units/ml penicillin and 100 µg/ml streptomycin, at 37℃ in a humidified atmosphere of 5% CO2.

**CCK-8 Cytotoxicity Assay**

The cell viability after drug treatment was assessed using CCK-8 assay as described previously[^10^](#_ENREF_10). Cells were harvested after trypsinization, resuspended in fresh culture medium, seeded into a 96-well plate at a density of 5,000 cells/well 24 hours prior to the experiments. Cells were washed, cultured in medium with treatment, incubated for 72 hours. Then 10 µl of CCK-8 solution were added to each well, incubated for 4 hours in a 37℃ incubator containing 5% CO2, and the absorbance was read at 570 nm. The IC50 was calculated from survival curves using the Bliss method[^11^](#_ENREF_11).

**Cell Apoptosis Assay**

Cells were harvested and washed twice with PBS, stained with Annexin V-FITC and propidium iodide (PI) in the binding buffer. Then the cells were incubated at room temperature in the dark and subjected to apoptosis assay by flow cytometry after 15 minutes. The early apoptotic cells and late apoptotic cells were quantified using the FlowJO software.

**Immunoblotting Assay**

Tumor tissues were grounded using liquid nitrogen. Cells or tumor tissues were lysed with radioimmunoprecipitation assay (RIPA) buffer complemented with protease inhibitors on ice for 15 minutes. The cell debris was removed by centrifugation, and proteins were then quantified using a BCA protein assay kit (Bio-Rad, CA) as previously described[^12^](#_ENREF_12). Protein samples were resolved by sodium dodecyl sulfate-polyacrylamide gel electrophoresis (10% SDS-PAGE) and transferred to polyvinylidene fluoride (PVDF) membrane. The membrane was blocked with 5% non-fat dried milk in Tris Buffered Saline-Tween (TBS-T) and then incubated with protein-specific primary antibodies overnight at 4℃. The membranes were washed using TBS-T, followed by incubation with a secondary antibody conjugated with horseradish peroxidase for two hours. Proteins were detected using enhanced chemiluminescence (ECL) detection reagents.

**Reverse Rranscription Polymerase Chain Reaction (RT-PCR)**

Total cellular RNA was isolated by Trizol reagent, and the cDNA was synthesized using oligo(dT)18 primers and M-MLV reverse transcriptase. cDNA was amplified with specific primers for CXCL8 and GAPDH. PCR was performed using the following primers: CXCL8, 5’-TAAATCTGGCAACCCTAGTC-3’(sense) and 5’-GCGTTCTAACTCATTATTCCGT-3’ (antisense); GAPDH, 5’-AATGCATCCTGCACCACCAACTGC-3’ (sense) and 5’-GGAGGCCATGTAGGCCATGAGGTC-3’ (antisense). The PCR products were separated by 1% agarose gels, stained with Biotium GelRed and visualized under UV; levels of mRNA were quantified using UVP VisionWorks LS Software.

**Enzyme Linked Immunosorbent Assay (ELISA) and immunohistochemical analysis**

The concentrations of CXCL8 in blood samples of ovarian cancer patients were analyzed using the Human CXCL8 ELISA kit according to the manufacturer’s protocol.

Immunohistochemistry was done as previously described[^12^](#_ENREF_12)^,^ [^13^](#_ENREF_13). Tumor samples were fixed, and paraffin embedded for further study. The slides were heat-immobilized according to antibody manufacturer’s instructions, monoclonal p-GSK-3β (Ser9), p-p70S6K (Thr389), Factor VIII antibodies were used for staining. The relative angiogenesis level was estimated by micro-vessel density (MVD) as described previously[^14^](#_ENREF_14)^,^ [^15^](#_ENREF_15).

**ROS Staining Assay**

For ROS staining, frozen sections were prepared and mounted on slides coated with 3-aminopropyltriethoxysilane and processed immediately. The intracellular ROS levels were detected using CM-H2DCFDA. CM-H2DCFDA was diffused into the cell and hydrolyzed by intracellular esterases to 2',7'-dichlorofluorescein, a non-fluorescent fluorescein analog, that could then be oxidized to highly fluorescent 2',7'-dichlorofluorescein by intracellular oxidants[^16^](#_ENREF_16). The sections were washed by Phosphate Buffer Saline (PBS) and incubated with 10 μM CM-H2DCFDA for 30 minutes. The sections were then washed by PBS and visualized under a fluorescence microscope.

**Orthotopic Tumor Model Using Ovarian Cancer Cells**

Four-week-old female nude mice (BALB/c-derived nu/nu) were purchased from the Shanghai Experimental Animal Center, maintained in a laminar-flow cabinet under specific pathogen-free conditions, and given standard food and water. All procedures involving animals were approved by the Institutional Committee on Animal Care of Zhengzhou University. Mice were kept under anesthesia during the entire procedure. Two 1 cm bilateral incisions were made at the back of the mice to expose ovaries, and 20 μl resuspended OVCAR-3 cells (1×10^6^ cells) infected by adenovirus and control were injected into the ovaries. After holding the site of injection for a minute with a cotton swab, the wounds were closed with surgical metal clips. 28 days after injection mice were sacrificed and tumor tissues and blood were collected. Tumors were trimmed out, weighed and sectioned into two parts, one was fixed in Bouin’s Fixative and paraffin embedded for immunohistochemical (IHC) staining, the other was snap frozen in liquid nitrogen and stored at -80℃ for further analysis. The vascularization was studied by determination of CD31 and hemoglobin content using the Drabkin’s method as previously described[^17^](#_ENREF_17).

**Adenovirus Preparation and Infection**

Recombinant adenoviruses were obtained using the AdEasy system[^18^](#_ENREF_18). Briefly, the gene of interest was subcloned into the shuttle vector pAdTrack-CMV, and the shuttle vector was linearized with *Pme*I. The linearized shuttle vector was cloned into a strain of BJ5183 bacterial cells that harbor the supercoiled backbone vector. Recombinants were selected with kanamycin, and the recombination was confirmed by restriction endonuclease analyses. The inverted terminal repeats of purified recombinant adenoviruses plasmid were exposed via *Pac*I, and the plasmid was transfected into AD-293 cells. The deleted viral assembly genes were complemented *in vivo*. A GSK-3β virus, an enzymatically inactive GSK-3β mutant containing a lysine-to-methionine substitution at residue 85 (GSK-3β (K85M)) virus, and a GFP protein virus were all derived from the same vector. Viral titer was checked by GFP, immunohistochemical staining, or by reading absorbance at 260 nm. Those viruses without GFP were titered using a BD Adeno-X Rapid Titer Kit. Adenoviruses overexpressing catalase, GPX, and p70S6K used in this study have been described previously[^19^](#_ENREF_19)^,^ [^20^](#_ENREF_20).

**Statistical Analysis**

Statistical analysis was performed via the SPSS software. All *in vitro* and *in vivo* data were presented as mean ± standard error (SE). Student’s unpaired t-test was used for basic statistical calculations. The correlation between CXCL8, ROS, p-GSK-3β (Ser9), and p-p70S6K (Thr389) expression, and the correlation between ROS and CXCL8 secretion were examined using Spearman’s correlation coefficient. The cutoff that maximized the sum of sensitivity and specificity for ROS and CXCL8 poor outcome of the disease was chosen using the standard ROC curve. The survival curves were estimated by Kaplan-Meier, and the resulting curves were compared using the log-rank test. Differences were considered statistically significant when the p-value (two-sided) was <0.05.
