# Supplementary material for: Reactive oxygen species mediate ovarian cancer development, platinum resistance, and angiogenesis via CXCL8 and GSK-3β/p70S6K1 axis
Source: Genes Dis. 2024 Jul 17;12(2):101378. doi: 10.1016/j.gendis.2024.101378 (PMC11629555; doi:10.1016/j.gendis.2024.101378)
Supplement: Multimedia component 2 [file mmc2.docx]

**Supplemental figure legends**

**Figure S1. Upregulation of endogenous ROS and serum CXCL8 were found in ovarian cancer which were correlated with poor survival rate of ovarian cancer patients.** (A) Quantitative analysis of the percentage of ROS-positive cells/field in cancer and normal samples. (B) Serum CXCL8 levels in normal control subjects and serous ovarian patients were measured and showed by histogram. (C) the level of CXCL8 was analyzed in ovarian cancer samples in GSE data base. (D) Serum CXCL8 level in ovarian cancer patients at cutoff 34.75 pg/ml. The x-axis denotes survival time after surgery and the y-axis denotes percentage of survivors. All data were shown as the means ± SE. *, **indicate the values are significantly different at *p*﹤0.05 and *p*﹤0.05, respectively.

**Figure S2. CXCL8 was upregulated in cancers and associated with poor prognosis**. The correlation between CXCL8 expression and prognosis in ovarian cancer analyzed by GSE database.

**Figure S3. ROS regulated CXCL8 expression via p-GSK-3β and p-p70S6K in ovarian cancer cells.** (A) A2780 and OVCAR-3 cells were starved for 24 hours and treated with indicated concentrations of H_2_O_2_. (B) A2780 and OVCAR-3 cells were infected by adenovirus carrying GFP or GSK-3β. The cells were treated with H_2_O_2_ (100 μM). (C) Cells were infected by adenovirus carrying GFP or GSK-3β (K85M) at 20 MOI (Multiplicity of Infection). (D) Cells were starved before incubation with LiCl (20 mM), an inhibitor of GSK-3β. (E) Cells were infected by adenovirus carrying GFP and p70S6K. RT-PCR was performed to detect CXCL8 and GAPDH levels. *indicates the value is significantly different at *p*<0.05 compared to the solvent control, # indicates the value is significantly different at *p*<0.05 compared to the GFP group.

**Figure S4. ROS regulated tumor growth and angiogenesis though CXCL8 induction, p70S6K activation, and GSK-3β inhibition *in vivo* using orthotopic ovarian cancer model.** OVCAR-3 cells were infected with adenovirus carrying catalase or GPX at 20 MOI, cells infected with or without adenovirus GFP were used as control. OVCAR-3 cells (1×10^6^ cells) were orthotopically injected into ovaries of nude mice (n=5). (A) Graphical representation of tumor weight (g). (B) The angiogenesis was analyzed by levels of hemoglobin. (C) Levels of CXCL8 protein in tumors were measured in duplicates. (D-F) Analysis of p-GSK-3β (Ser9), p-p70S6K (Thr389) and CXCL8 protein levels in each tumor. The quantitative levels of target molecules were analyzed using software and represented as mean ± SEM. * indicates the value is significantly different at *p*<0.05 compared to the solvent control, # indicates the value is significantly different at *p*<0.05 compared to the GFP group (*n* = 5).

**Figure S5. GSK-3β was an essential downstream target of ROS to mediate tumor growth and angiogenesis.** OVCAR-3 cells were infected with adenovirus carrying GSK-3β, GSK-3β K85M or GFP control at 20 MOI. OVCAR-3 cells (1×10^6^ cells) were orthotopically injected into ovaries of nude mice. The mice were euthanized on Day 28. (A) Graphical representation of tumor weight (g). (B) The relative angiogenesis was analyzed by the levels of hemoglobin. (C) Quantification analysis of MVD in the tumor sections was performed. The graph represents the mean ± SEM from five different tumor sections. (D) The protein expression of CXCL8 in tumor tissues were analyzed by immunoblotting.

**Figure S6. The expression of p-GSK-3β (Ser9), p-p70S6K (Thr389) and CXCL8 were increased in ovarian cancer tissues.** (A) Total protein extracts of the human ovarian cancer tumor samples and normal ovary samples were obtained and analyzed the protein expression by Western blotting assay. (B) Ovarian cancer tissues were embedded in paraffin and tested for CXCL8, p-GSK-3β (Ser9), and p-p70S6K (Thr389) expression using immunohistochemistry. Graphs illustrated the correlation between (C) CXCL8 expression and circulating CXCL8 level, (D) CXCL8 expression and p-p70S6K (Thr389), (E) CXCL8 expression and p-GSK-3β (Ser9) in ovarian cancer tissues.

**Figure S7. p70S6K1 inhibitor PF-4708671 had significantly synergistic effect with** **cis-platinum or carboplatin in human ovarian cancer cells.** (A) OVCAR3 cells were seeded into 96 well plate, and treated cells with indicated concentration of PF-4708671 and cis-platinum/carboplatin, and cell survival was measured by CCK-8 assay after 72 hours. Data were analyzed with CompuSyn software, the scattergram of combined effects were shown. (C) The cell apoptosis results were displayed using a bar chart. The proportions of Annexin V+/PI- and Annexin V+/PI+ cells indicated the early and late stage of apoptosis. The concentrations of each agent were used as follows: cis-platinum 30 μM and PF-4708671 100 μM in OVCAR3 cells, cis-platinum 30 μM and PF-4708671 3 μM in A2780 cells. **,***indicate the values are significantly different at *p*﹤0.01 and *p*﹤0.001, respectively.
