# Supplementary material for: Reactive oxygen species mediate ovarian cancer development, platinum resistance, and angiogenesis via CXCL8 and GSK-3β/p70S6K1 axis
Source: Genes Dis. 2024 Jul 17;12(2):101378. doi: 10.1016/j.gendis.2024.101378 (PMC11629555; doi:10.1016/j.gendis.2024.101378)
Supplement: Multimedia component 3 [file mmc3.pdf]

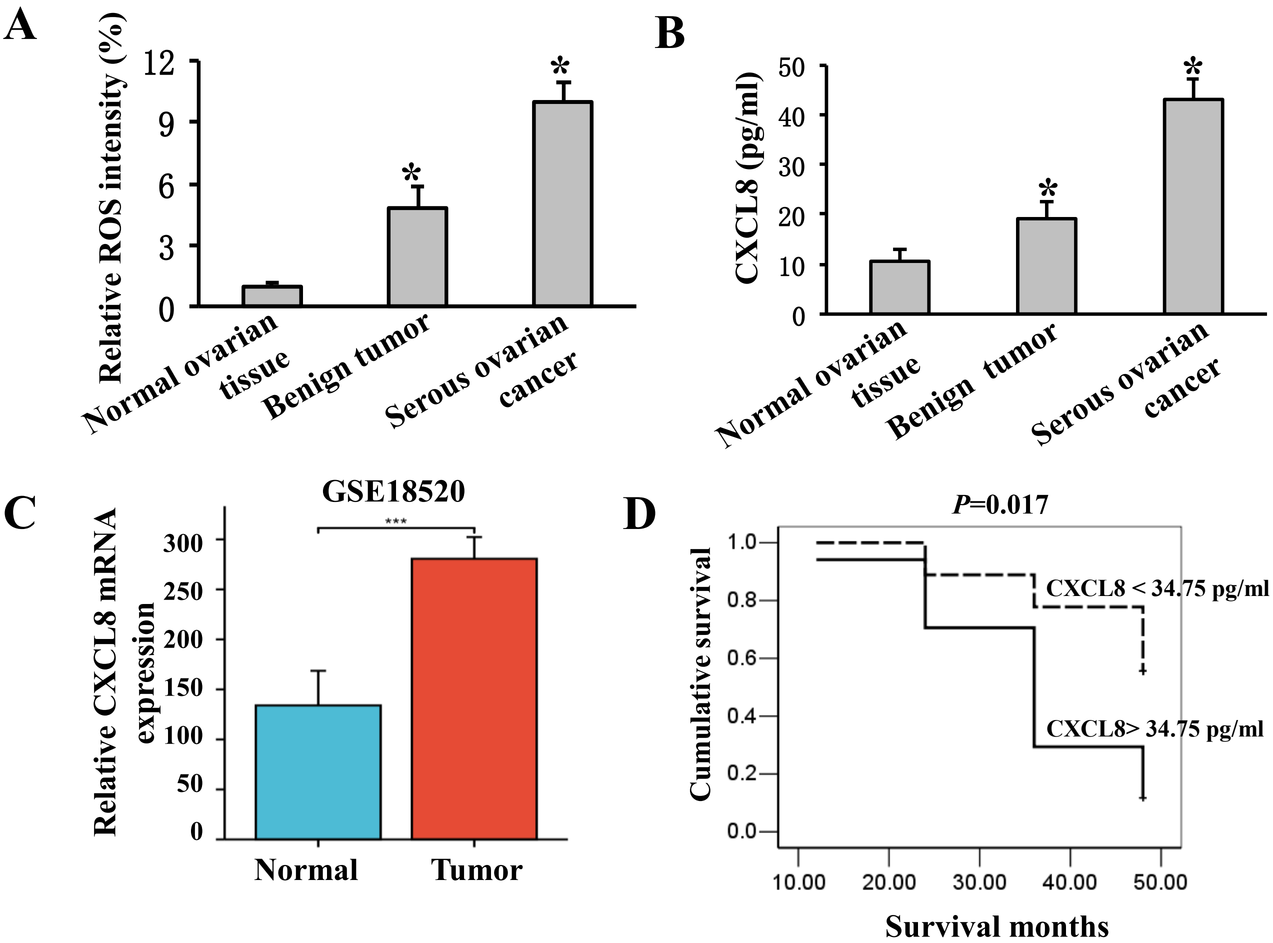

Supplementary Figure 1

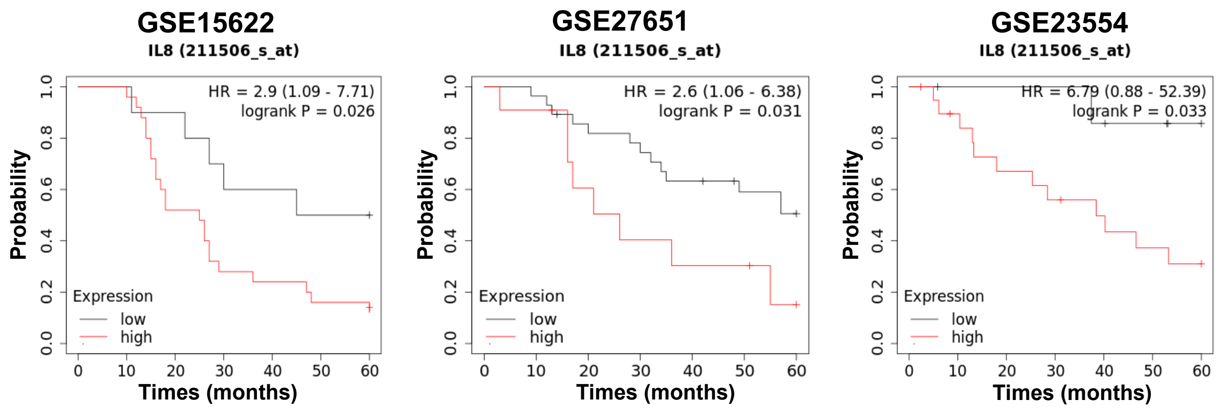

Supplementary Figure 2

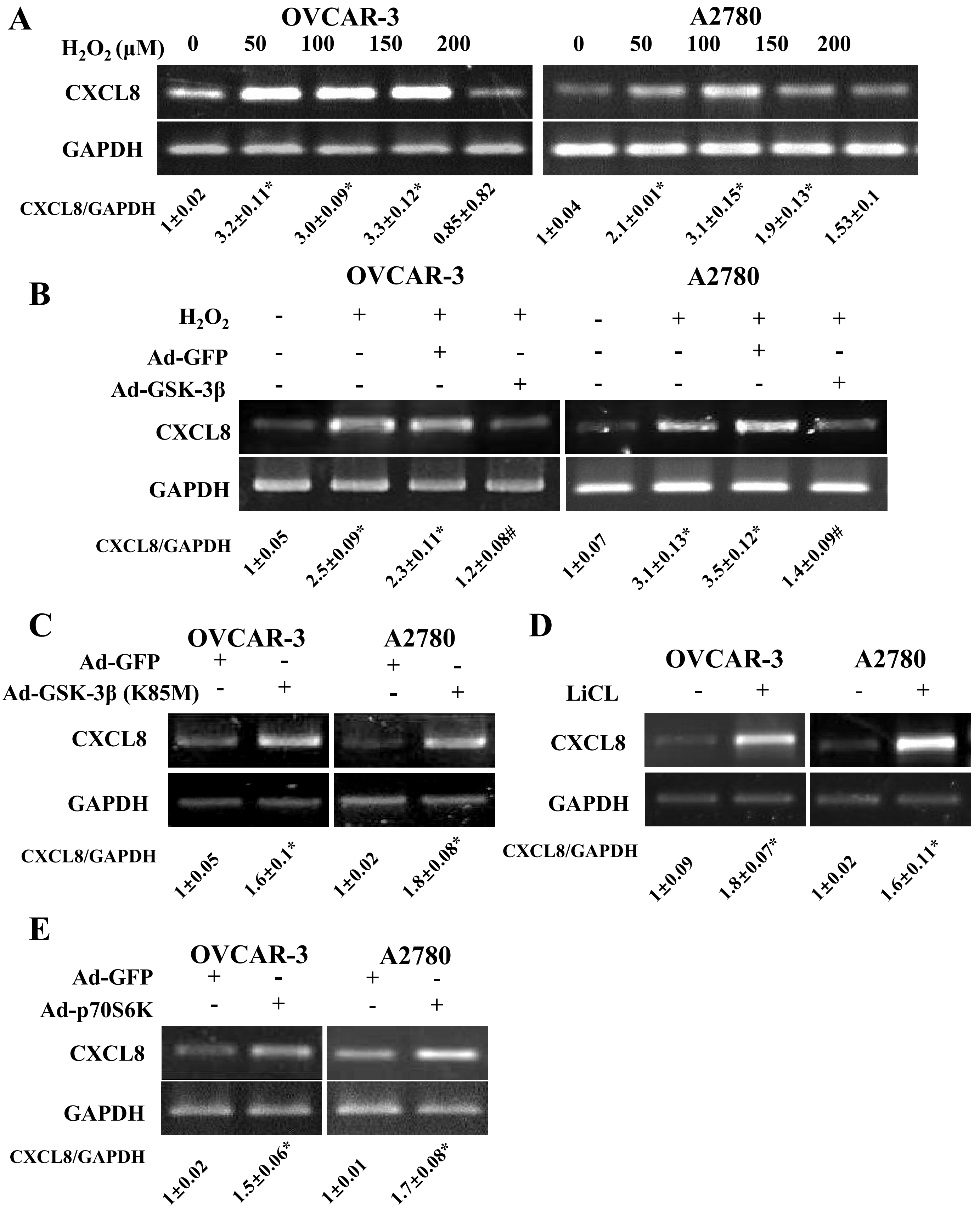

Supplementary Figure 3

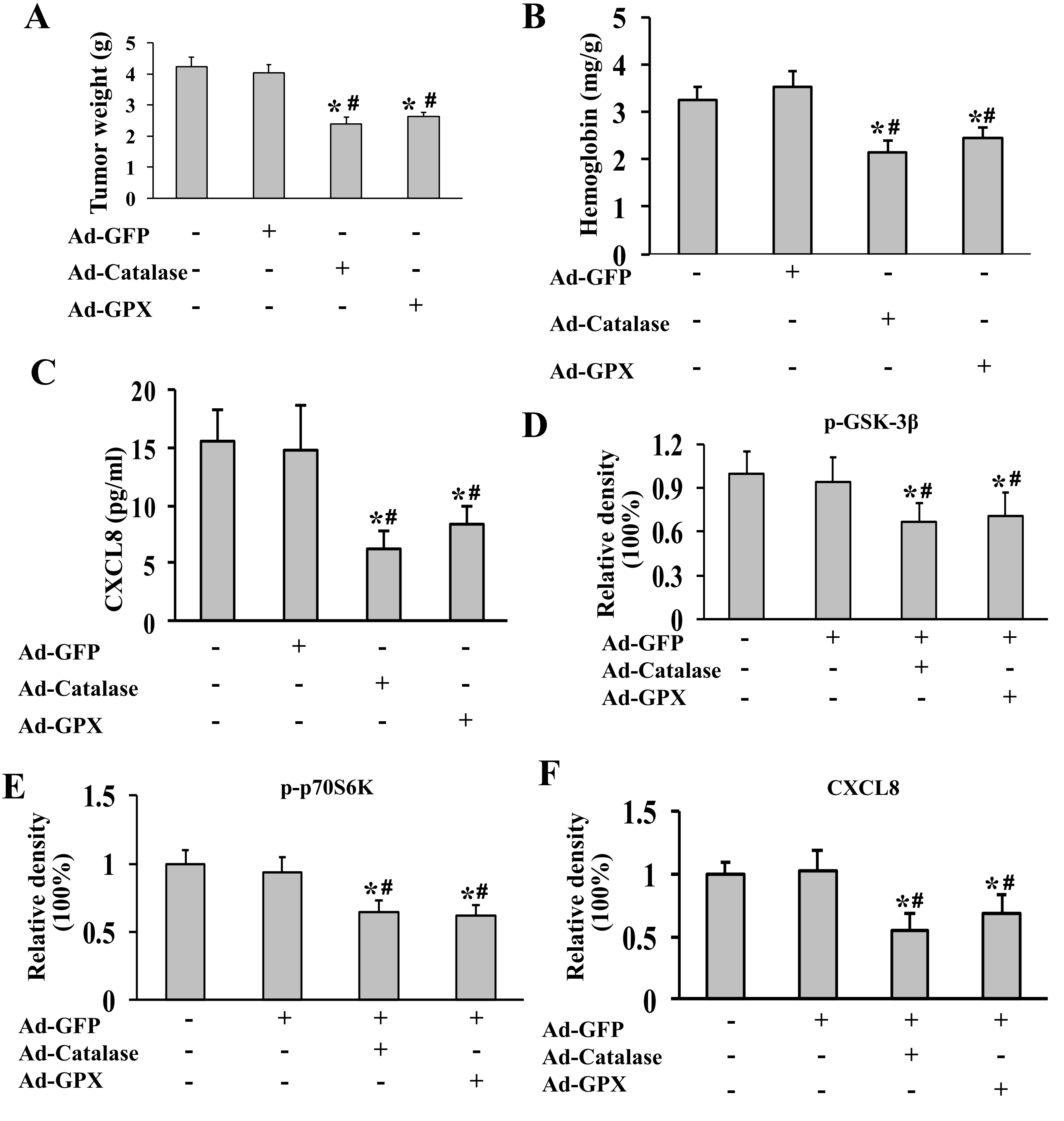

Supplementary Figure 4

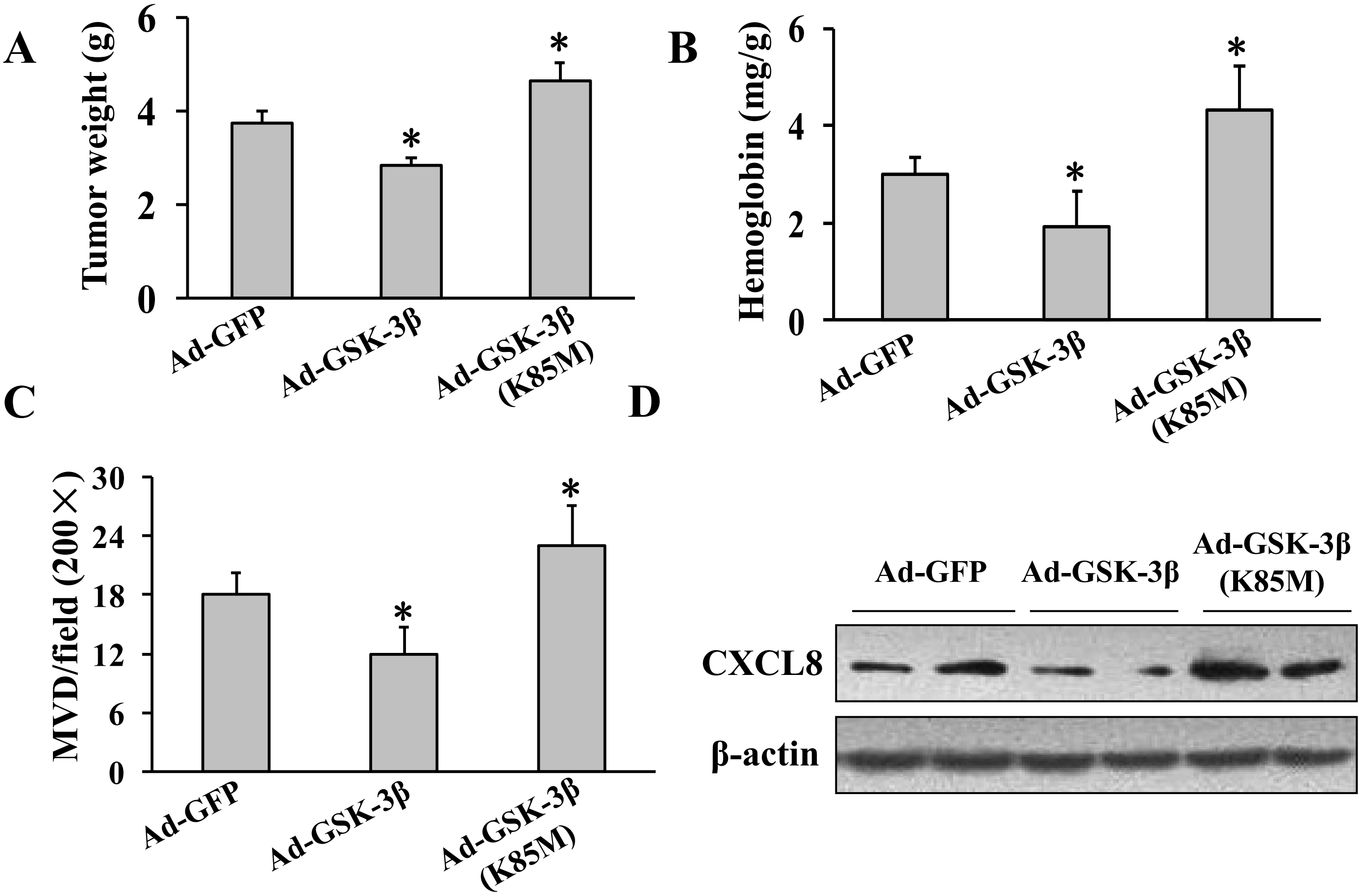

Supplementary Figure 5

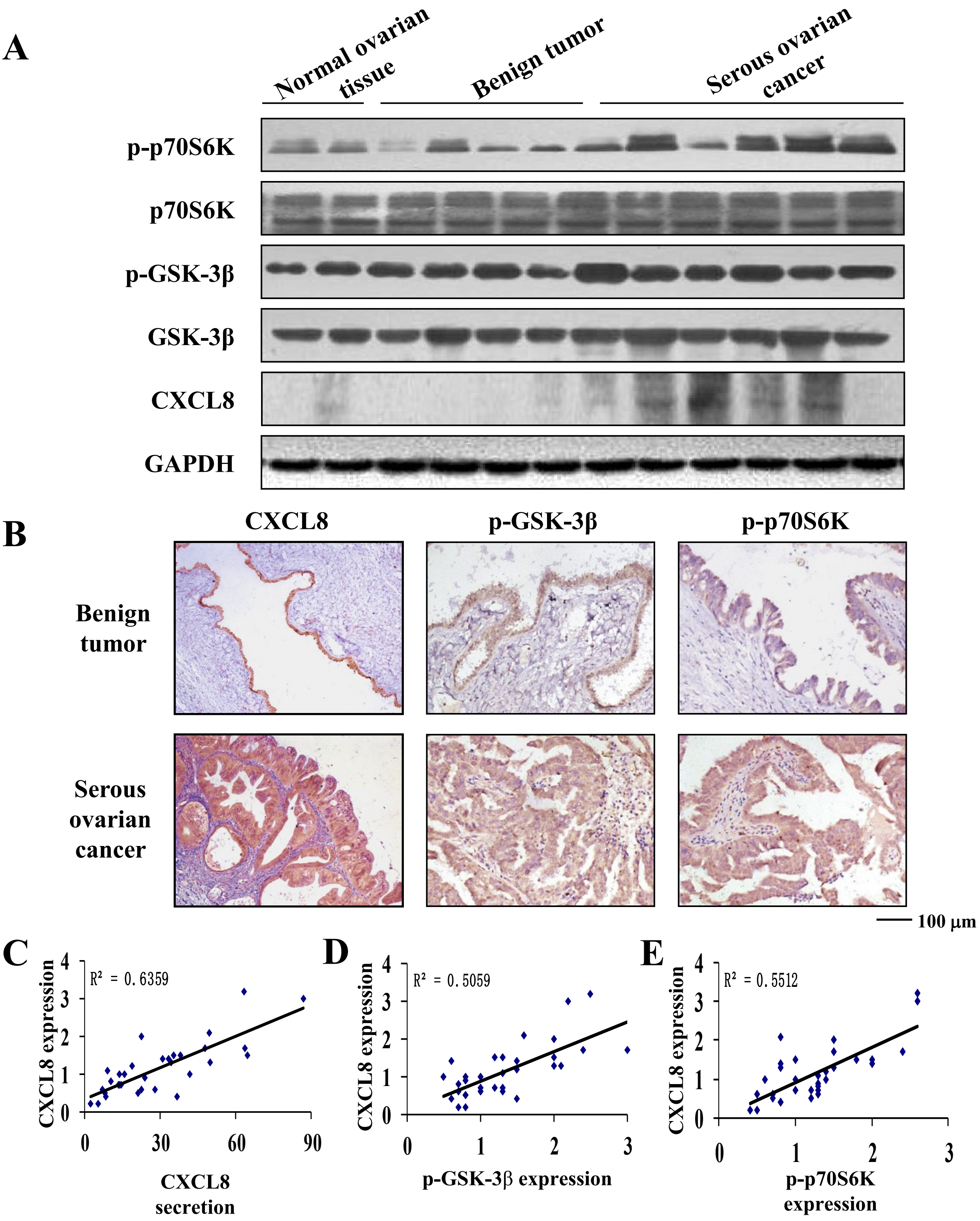

Supplementary Figure 6

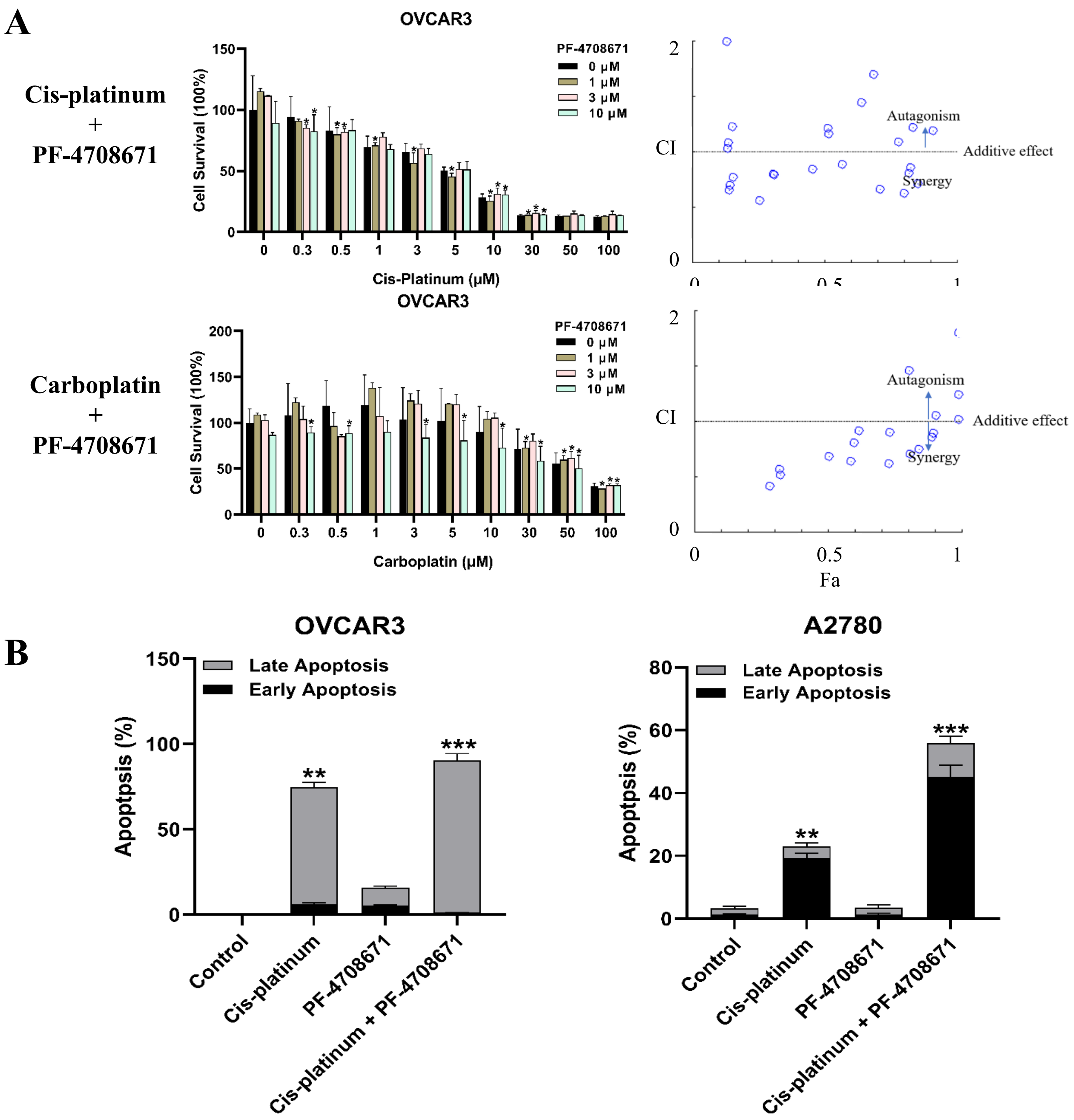

Supplementary Figure 7
